# Echocardiographic Findings in Patients with Atrial Fibrillation in a Tertiary Care Center of Nepal: A Descriptive Cross-sectional Study

**DOI:** 10.31729/jnma.5408

**Published:** 2021-01-31

**Authors:** Smriti Shakya, Ratna Mani Gajurel, Chandra Mani Poudel, Hemant Shrestha, Surya Devkota, Sanjeev Thapa

**Affiliations:** 1 Department of Cardiology, Manmohan Cardio-Thoracic Vascular and Transplant Centre (MCVTC), Institute of Medicine, TUTH, Kathmandu, Nepal

**Keywords:** *atrial fibrillation*, *left atrium*, *mitral valve*, *rheumatic heart disease*, *thrombus*

## Abstract

**Introduction:**

Atrial fibrillation is the most prevalent supraventricular arrhythmia responsible for the large morbidity and mortality burden worldwide. There are various causes of atrial fibrillation that may affect the prognosis of patients. This study was intended to determine different echocardiographic findings in patients with atrial fibrillation in a tertiary care center.

**Methods:**

A descriptive cross-sectional study was conducted at Manmohan Cardiothoracic Vascular and Transplant Center, Institute of Medicine, among 175 patients with atrial fibrillation admitted in the cardiology department from June 2017 to October 2018. It was approved by the Institutional Review Board of the Institute of Medicine (Ref.:411(6-11-E)2/073/074). Convenience sampling was used. Statistical analysis was done using Statistical Package for Social Sciences version 21.0.

**Results:**

A total of 175 patients with atrial fibrillation were enrolled where Rheumatic heart disease 68 (38.9%) was the leading cause in which 54 (79.4%) had mitral valve lesion, 1 (1.5%) had aortic valve lesion and rest had a combination of both. The mixed lesion of mitral stenosis and mitral regurgitation was the commonest. The left atrium size was larger in valvular atrial fibrillation (47.29±6.651mm). The left ventricular systolic dysfunction was seen more in non-valvular atrial fibrillation. The commonest site of thrombus formation was left atrium 7 (63.6%).

**Conclusions:**

Atrial fibrillation was common in rheumatic heart disease, especially mixed lesions of mitral stenosis and regurgitation. Valvular atrial fibrillation had a larger left atrium. The thrombus was seen in mitral stenosis and left ventricular systolic dysfunction. The left atrium size and left ventricular ejection fraction were associated with the occurrence of atrial fibrillation.

## INTRODUCTION

Atrial fibrillation (AF) is a common supraventricular tachyarrhythmia affecting 1% to 2% of the general population.^1^ The risk of AF generally increases with aging, hypertension, coronary artery disease, diabetes mellitus, alcohol use.^2,3^ However, the valvular disease is the most common substrate for AF in areas with a high prevalence of rheumatic heart disease and is a risk factor for embolic stroke and heart failure.^4,5^

The cardiac remodeling that occurs in response to various causes tends to increase the left atrial pressure and size and alter wall stress creating a substrate to cause AF.^6,7,8^ The enlarged left atrium (LA) has been correlated with AF occurrence and cardiovascular events.^9,10^ The left ventricular ejection fraction (LVEF) is also associated with AF development and its consequences.^11,12^

This study is aimed to determine different echocardiographic findings in patients with atrial fibrillation in a tertiary care center.

## METHODS

A descriptive cross-sectional study was carried out in the Department of Cardiology of Manmohan Cardiothoracic Vascular and Transplant Center, Institute of Medicine, Kathmandu, Nepal, from June 2017 to October 2018. All the patients with atrial fibrillation who presented to the department of cardiology were enrolled in the study. It was approved by the Institutional Review Board of the Institute of Medicine (Ref.: 411(6-11-E)2/073/074).

The sample size was calculated using the formula:

$$\begin{array}{ll} \text{n} & ={\text{Z}}^{\text{2}}\times \text{p}\times \text{q}/{\text{e}}^{\text{2}}\\ & ={\left(1.96\right)}^{2}\times 0.1\times 0.9/{\left(0.05\right)}^{\text{2}}\\ & =139 \end{array}$$

Where,

n = required sample size
Z = 1.96 at 95% confidence interval
p = prevalence
q = 1-p
e = margin of error, 5%

Adding a 10% non-responsive rate, the sample size was estimated to be 154. However, the total sample size taken was 175.

With the written informed consent, a detailed history was taken, and a physical examination was done. Twelve-lead electrocardiography (ECG) was taken and transthoracic echocardiography with GE. Vivid seven was done to look for left atrium (LA) size, LVEF, and left ventricular wall thickness assessed in the parasternal long-axis view. The LA dimension of more than 40mm was considered dilated. The septal and posterior left ventricular wall was measured and considered abnormal if ≥10mm in females and ≥11mm in males. The LV function was calculated by M-mode or Simpson's method and was considered abnormal if below 55 percent; however severe LV systolic dysfunction was considered if LVEF was less than 30 percent. The valvular morphology, stenotic or regurgitant lesions, clots or any other defects were also noted. We used the Statistical Package for Social Sciences version 21.0 for statistical analysis. The data were presented in number and percentage. Categorical variables were reported as percentages. Continuous variables were reported as mean with standard deviation.

## RESULTS

Among 175 total patients with Atrial Fibrillation, the mean age of study patients was 60±16.79 years, ranging from 15 years to 92. Among valvular AF, the mean age was 47.9±14.013, and among non-valvular AF, it was 67.85±13.507 years. The female patients were predominant, i.e., 98 (56%) whereas 77 (44%) were male patients. The female patients seemed to suffer AF at an earlier age (57.17±17.84 years) than male patients (63.82±14.63 years).

We found various etiologic factors of AF among which the majority 68 (38.9%) had Rheumatic heart disease, 29 (16.6%) had Dilated Cardiomyopathy (DCM), 10 (5.7%) had congenital heart disease (included Atrial Septal Defect Ostium Secundum type, Ventricular Septal Defect and Pulmonary Stenosis), 13 (7.4%) had coronary artery disease (STEMI or NSTEMI), 5 (2.9%) had ischemic cardiomyopathy, 17 (9.7%) had hypertension only (in 16 patients hypertension was associated with other diseases), 16 (9.1%) had degenerative valvular heart disease (DVHD), 2 (1.1%) had mitral valve prolapse (MVP), 2 (1.1%) had hypertrophic cardiomyopathy, 8 (4.6%) had miscellaneous causes [such as alcohol binge drinking, left atrial myxoma, myocarditis, Wolff Parkinson White syndrome (WPW), sick sinus syndrome, hyperthyroidism and chronic obstructive pulmonary disease] and 5 (2.9%) were idiopathic.

The patients with various etiologies presented to the hospital with varied causes mentioned in Table 1.

**Table 1:** Causes of hospital admission in patients with AF.

Cause of hospital admission	No. of patients n (%)
Heart failure	50 (28.57)
AF with Fast ventricular rate	22 (12.57)
Warfarin induced coagulopathy	16 (9.14)
Coronary artery disease	14 (8.00)
Pneumonia	14 (8.00)
Thromboembolism	11 (6.29)
Hypertension	8 (4.57)
Chronic obstructive pulmonary disease	7 (4.00)
Percutaneous Mitral Valvotomy	7 (4.00)
Cardiogenic shock	6 (3.43)
Right heart failure	6 (3.43)
Infective endocarditis	3 (1.71)
Bradycardia	3 (1.71)
Pre-operative coronary angiography	3 (1.71)
Urosepsis	2 (1.14)
Percutaneous pulmonary valvuloplasty	1 (0.57)
Thyroid storm	1 (0.57)
Deep vein thrombosis	1 (0.57)
Total	175

Among rheumatic valvular diseases, the mitral valve disease 54 (79.4%) was more associated with atrial fibrillation than aortic valve disease 1 (1.5%), the rest were both. The commonest was a mixed lesion of mitral stenosis and mitral regurgitation 23 (33.8%) followed by mitral stenosis 20 (29.4%) then mitral regurgitation 8 (11.8%). There were 10 (14.7%) patients who had only a mitral valve replaced (MVR), 1 (1.5%) patient had an only aortic valve replaced (AVR) and 6 (8.8%) patients had both the valves replaced. Four patients with aortic stenosis and three patients of aortic regurgitation were seen in combination with mitral valve lesions. The mitral regurgitation was seen in many other conditions in addition to rheumatic causes 27 (32.9%) such as DVHD 13 (15.8%), DCM 12 (14.6%), hypertension 10 (12.2%), coronary artery disease 8 (9.8%), ischemic cardiomyopathy 4 (4.9%), MVP 2 (2.5%) and other miscellaneous causes.

The mean left atrium size in valvular AF was 47.29±6.651mm with a minimum LA diameter of 37mm and a maximum of 73mm. The mean left atrium size in nonvalvular AF was 42.20±4.356mm with a minimum of 33mm and a maximum of 59mm.

Left ventricular hypertrophy (LVH) was observed in 19 (10.86%) patients. It was seen in conditions such as systemic hypertension, hypertrophic cardiomyopathy, aortic stenosis and ventricular septal defect.

About 88 (50%) patients had normal LV function, while mild and moderate LV systolic dysfunction was present in 23 (13%) patients each and 41 (24%) had severe LV systolic dysfunction. There was more LV systolic dysfunction in the NVAF group than the valvular one. Table 2 shows the distribution of left ventricular systolic function among valvular and nonvalvular AF.

**Table 2:** LV systolic function among valvular and non valvular AF.

LV systolic function	Valvular AF n (%)	Nonvalvular AF n (%)	Total no. of patients n (%)
Normal	42 (47.7)	46 (52.3)	88 (50.3)
Mild LVSD	9 (39.1)	14 (60.9)	23 (13.1)
Moderate LVSD	10 (43.5)	13 (56.5)	23 (13.1)
Severe LVSD	7 (17)	34 (83)	41 (23.5)
Total	68	107	175 (100)

The median LA size and LVEF was higher in valvular AF group compared to non-valvular AF (Table 3).

**Table 3:** Description of LA size, LVEF and LVH in valvular and non-valvular AF.

Variables	Valvular Afib (n = 68)	Non-valvular Afib (n = 107)
LVH present, n (%)	0 (0.0)	19 (17.8)
LA size (mm)	47.2 (37.0 - 73.0)	42.2 (33.0 - 59.0)
LVEF (%)	55 (15 - 70)	45 (15 - 65)

Among total intracavitary clots, the mitral stenosis was associated with 64% of it, including a clot in the left atrium proper (37%) and both left atrium and left atrial appendage (27%). The patients with mitral valve replacement (9%), double valve replacement (9%) and acute coronary syndrome (9%) had left ventricular clot, and a case of dilated cardiomyopathy (9%) had left atrium and ventricular clot (Table no. 4).

**Table 4:** Formation of clots in cardiac chambers in different conditions.

Underlying disorders	Location of clots		Mitral regurgitation	LVEF(%)	No. of patients n (%)
	Left atrium	LA and LAA		
Moderate MS	1		Trace	55	
Severe MS	1		Mild	60	
Moderate MS	1		Severe	60	7 (64)
Severe MS		1	Mild	45
Severe MS		1	Mild	50	
Severe MS		1	Moderate	65	
Severe MS		1	Mild	55	
DVR	LV		Trace	20	1 (9)
ACS	LV		Moderate	20	1 (9)
DCM	LA, LV		Moderate	30	1 (9)
MVR	LV		Trace	15	1 (9)
Total					11 (100)

## DISCUSSION

The increase in the prevalence of atrial fibrillation and its significant consumption of health resources globally raises concern regarding the knowledge of multiple associated factors about its causation and consequences. Henceforth the various chief echocardiographic features associated with AF were studied in this study.

Rheumatic heart disease (38.9%) was seen as the commonest cause of atrial fibrillation. It was shown that among total rheumatic heart disease, rheumatic mitral valve disease (79.4%) was associated with AF more than other rheumatic valve diseases. The mitral regurgitation was seen in many conditions apart from RHD, such as dilated cardiomyopathy, ischemic cardiomyopathy, MVP, hypertrophic cardiomyopathy, degenerative valvular heart disease and myocardial infarction. But considering rheumatic valvular lesions, the mixed lesion of mitral stenosis and mitral regurgitation (33.8%) was commonest followed by mitral stenosis (29.4%) and then mitral regurgitation (11.8%). This finding was similar to a study done by Andrade et al.^13^ The mitral valve disease caused a large left atrium and elevated left atrial pressure, which increases the dispersion of refractoriness and increased automaticity, creating the milieu for initiation and perpetuation of AF.^14^ The fibrosis and degeneration of the atrial myocardium in valvular heart disease, especially rheumatic etiology, disturb impulse propagation in the atria leading to AF that persists even after valve replacement or repair.^15,16^

Our study showed the mean left atrium size was 47.3mm in valvular AF and 42.2mm in NVAF. The median LA size was higher in valvular AF than NVAF. The largest LA size was seen in severe rheumatic mitral regurgitation, which was 73mm. The finding was similar in some other studies.^17,18^ The LA enlargement was the echocardiographic parameter that was most associated with AF.^11^

The left ventricular hypertrophy was seen in 10.86% of patients. The prevalence of LVH was higher in patients with non-valvular AF, especially hypertension. The left ventricular hypertrophy leads to impaired left ventricular diastolic filling, increased left atrial pressure, left atrial enlargement, increased atrial fibrosis leading to increased atrial ectopic activity, and slowing of intra- and interatrial electrical conduction velocities.^19^

The other parameter studied was left ventricular systolic function, which showed that about 50% of patients had LV systolic dysfunction, in which 26% had mild to moderate LV systolic dysfunction, and 24% had severe LV systolic dysfunction. Most of the LV systolic dysfunction was seen in patients with NVAF than valvular AF. It included patients mostly with DCM, ischemic cardiomyopathy, coronary artery disease and degenerative valvular diseases. A study revealed that a low LVEF was the second most associated parameter for the occurrence of AF.^11^

In our study, the clots were seen more in the left atrium and left atrial appendage in patients with mitral stenosis than mitral regurgitation as the high-velocity flow inside the left atrium in case of mitral regurgitation may prevent thrombus formation.^20^ Another common site for thrombus formation was left ventricle which was seen in patients with LV systolic dysfunction in cases of dilated cardiomyopathy, acute coronary syndrome and mitral and double valve replacement due to reduced wall motion and stasis of blood in ventricle forming thrombus similar to the findings in a study done by Talle et al.^21^

There are some limitations to this study. This is a single-center study through a tertiary care super-specialty center. Some variables could not be considered, such as left ventricular diastolic function, left atrial volume. Rather a practically employed left atrial diameter was taken. Since transesophageal echocardiography wasn't possible in all cases, a left atrial appendage clot may have been missed in some cases.

## CONCLUSIONS

Rheumatic heart disease is the commonest cause of AF in developing countries. AF is commonly seen in mitral valve disease, in which mixed lesion of mitral stenosis and regurgitation is the most common. The left atrial size and left ventricular systolic dysfunction are the markers for the development of AF. The thrombus is seen in the left atrium in mitral stenosis and the left ventricle in cases with left ventricular systolic dysfunction.
